# Supplementary material for: From sequence to enzyme mechanism using multi-label machine learning
Source: BMC Bioinformatics. 2014 May 19;15:150. doi: 10.1186/1471-2105-15-150 (PMC4229970; doi:10.1186/1471-2105-15-150)
Supplement: Additional file 2 — Java code of ml2db. Additional file ml2db_code.tar.gz contains the Java source code to run the multi-label machine learning experiments and save the results to database. The code’s Javadoc is included. [file 1471-2105-15-150-S2.zip › additional file 2/ml2db/utils/doc/uk/ac/ed/inf/utils/database/TableManager.html]

TableManager


---


|  |  |  |
| --- | --- | --- |
| **Overview** \| **Package** \| **Class** \| **Use** \| **Tree** \| **Deprecated** \| **Index** \| **Help** | |  |
| **PREV CLASS**   **NEXT CLASS** | **FRAMES**    **NO FRAMES**     **All Classes** |  |
| SUMMARY: NESTED \| FIELD \| CONSTR \| METHOD | DETAIL: FIELD \| CONSTR \| METHOD |  |


---


## uk.ac.ed.inf.utils.database Class TableManager

```
java.lang.Object
  uk.ac.ed.inf.utils.database.DbManaged
      uk.ac.ed.inf.utils.database.TableManager
```

---

``` public class TableManager extends DbManaged ```

Class to create/read/write to a database table.

**Version:**
:   10 Jun 2010

**Author:**
:   Luna De Ferrari luna.deferrari-at-ed.ac.uk

---

| **Field Summary** | |
| --- | --- |
| `TableWriter` | `m_writer`             the database recorder |


**Constructor Summary**

`TableManager(DbManager dbManager,
java.lang.String tableName)`
  
          Constructor to create a generic manager to read/write to a table.

`TableManager(DbManager dbManager,
Table table)`
  
          Constructor to create a manager to create, read and write a table.


| **Method Summary** | |
| --- | --- |
| `Table` | `getTable()` |
| `TableCreator` | `getTableCreator()` |
| `java.lang.String` | `getTableName()` |
| `TableReader` | `getTableReader()` |
| `TableWriter` | `getTableWriter()` |

**Methods inherited from class uk.ac.ed.inf.utils.database.DbManaged**

`getDbConnection, getDbManager`

**Methods inherited from class java.lang.Object**

`equals, getClass, hashCode, notify, notifyAll, toString, wait, wait, wait`

**Field Detail**

### m\_writer

```
public TableWriter m_writer
```

:   the database recorder


**Constructor Detail**

### TableManager

```
public TableManager(DbManager dbManager,
                    java.lang.String tableName)
```

:   Constructor to create a generic manager to read/write to a table.

    **Parameters:**: `dbManager` -: `tableName` -

---


### TableManager

```
public TableManager(DbManager dbManager,
                    Table table)
```

:   Constructor to create a manager to create, read and write a table.

    **Parameters:**: `dbManager` -: `table` -


**Method Detail**

### getTable

```
public Table getTable()
```

---


### getTableCreator

```
public TableCreator getTableCreator()
```

---


### getTableName

```
public java.lang.String getTableName()
```

---


### getTableReader

```
public TableReader getTableReader()
```

---


### getTableWriter

```
public TableWriter getTableWriter()
```


---


|  |  |  |
| --- | --- | --- |
| **Overview** \| **Package** \| **Class** \| **Use** \| **Tree** \| **Deprecated** \| **Index** \| **Help** | |  |
| **PREV CLASS**   **NEXT CLASS** | **FRAMES**    **NO FRAMES**     **All Classes** |  |
| SUMMARY: NESTED \| FIELD \| CONSTR \| METHOD | DETAIL: FIELD \| CONSTR \| METHOD |  |


---
